# Advancing biomedical data analytics using explainable neural network-based learning model for progressive neurodegenerative disorder diagnosis

**DOI:** 10.1038/s41598-026-57800-y

**Published:** 2026-06-13

**Authors:** S. Praveena, E. Laxmi Lydia, Suresh Betam, N. Rahul Pal, Sivanagaraju Vallabhuni, Vonteru Srikanth Reddy, Shreyas Rajendra Hole

**Affiliations:** 1 Electronic and Communication Engineering, Mahatma Gandhi Institute of Technology, Gandipet, Hyderabad, Telangana India; 2 Department of Computer Science and Engineering, Vignan’s Institute of Engineering for Women, Visakhapatnam, Andhra Pradesh 530046 India; 3 Department of CSE, Koneru Lakshmaiah Education Foundation, Vaddeswaram, Guntur, Andhra Pradesh India; 4 Dept of AIML, Aditya University, Aditya Nagar, Surampalem, Andhra Pradesh 533437 India; 5 Computer Science & Engineering (AI&ML), Lakireddy Bali Reddy College of Engineering (Autonomous), L.B. Reddy Nagar, NTR Dist., Mylavaram, Andhra Pradesh 521230 India; 6 Department of Computer Science and Engineering, VNR - Vignana Jyothi Institute of Engineering &Technology, Hyderabad, Telangana 500118 India; 7 Department of Computer Science and Engineering, Symbiosis Institute of Technology Nagpur Campus Symbiosis International (Deem University), Pune, India

**Keywords:** Huntington’s disease, Cycle-Norm-Adam optimizer, Deep learning, Hybrid feature selection, Explainable artificial intelligence, Computational biology and bioinformatics, Diseases, Mathematics and computing, Neurology, Neuroscience

## Abstract

Huntington’s disease (HD) is an inherited neurological disease caused by variations in the huntingtin (HTT) gene, which leads to neuronal degeneration. Conventionally, HD is affiliated with the gathering and misfolding of mutant HTT arising from an increased number of CAG triplets. Artificial Intelligence has emerged as an important tool in healthcare, supporting the monitoring, detection, and management of HD. Machine learning and deep learning methods are widely used for automated HD identification using neuroimaging, genetic, and clinical data. However, most DL models behave like a black box, making it difficult to interpret decision-making from clinical data, which reduces trust in medical applications. Therefore, this study presents an Explainable Neural Network-Driven Learning Model for Neurodegenerative Disorder Diagnosis (XNNLM-NDD). The primary objective of the proposed model is to examine clinical attributes and identify disease patterns efficiently for precise diagnosis. The model performs feature selection using a hybrid combination of minimum redundancy maximum relevance and ReliefF methods to select the most informative and non-redundant features from the dataset. For classification, the proposed approach employs a feature tokenizer-transformer model, which can capture complex feature interactions and improve classification accuracy on structured medical data. Furthermore, the model is optimized using the Cycle-Norm-Adam algorithm. For ensuring model transparency and interpretability, SHAP-based explainable artificial intelligence method is used to highlight the contribution of each feature towards the final prediction. The experimental evaluation is carried out on the Huntington Disease Dataset sourced from Kaggle. The results show that the proposed XNNLM-NDD approach accomplishes improved performance with an accuracy of 96.50% compared to existing techniques, indicating its efficiency in progressive neurodegenerative disorder diagnosis.

## Introduction

Huntington’s disease (HD) is an intensely affecting neurodegenerative disease that not only impacts individuals but also casts a prolonged shadow over their households. It portrays a compound medical image, denoted by the inexorable development of psychiatric symptoms, cognitive decline, and motor dysfunction, eventually concluding in intellectual disability and, unfortunately, shortening the person’s life-time^1^. The significance of HD has caused rising concern amongst the research and medical communities globally, provoking versatile efforts to not only untangle its pathophysiological and etiological complexities, but also begin with progressions in its initial identification and organization^2^. Surveys in other neurodegenerative illnesses like Parkinson’s and Alzheimer’s have correspondingly aimed to decipher their complex mechanisms, which leads to progress in interpreting their pathology and pave the way for potential therapy advances^3^. The poly-Q extension is because of the irregular CAG trinucleotide recurrences in the mutant HTT (mHTT). HTT protein is mostly produced in brain neurons, where it is found throughout the cytoplasm and attached to vesicular membranes^4^. In recent times, artificial intelligence (AI) approaches play a vital role in evolving the management and analysis of HD because of the intricacy, progressive, and variable nature of the disease^5^. Likewise, these methods, especially applied on huge, multidimensional datasets, can provide substantial advancements by precisely analyzing distinct variety of data^6^^,^^7^ Not merely, but also carry the severity levels up by their ability to detect delicate patterns in information, which may be complex for physicians to identify, empowering initial diagnosis, accurate phenotyping, and best observation of illness development^8^. Moreover, AI can support these diverse diagnostic modalities, supplying a more global perspective of the disorder and its varied manifestations across individuals.

In addition, HD upgrades eventually, AI tools play an important role in controlling situation transforms, forecasting health prognosis, and conclusively managing personalized therapy models, which are essential for enhancing quality of life and patient care^9^. Specifically, in recent years, there has been a rising attention in utilizing AI to help in the analysis of HD and its many conditions, the ability to render several data types, and for the identification of key biomarkers. Though these trends, an extensive source of reference, enclosing the depth of machine learning (ML) and AI usage in HD diagnosing, have lacked. AI techniques have been increasingly used in neurodegenerative disorder analysis to support disease classification, medical image interpretation, biomarker identification, progression prediction, and clinical decision-making^10^. Machine learning and deep learning approaches help analyze complex biomedical data and identify hidden neurological patterns that may not be easily detected through traditional clinical methods. These advancements have improved diagnostic accuracy and enabled more efficient monitoring of disease progression in disorders such as Huntington’s disease, Alzheimer’s disease, and Parkinson’s disease^11^. Explainable Artificial Intelligence (XAI) aims to highlights these challenges by clearly explaining how the models are reaching their significance. By enhancing interpretability, XAI support to moderate ethical and medical issues and promotes better trust in AI-driven healthcare techniques^12^. Techniques like feature attribution recognize the Electroencephalography features, which most affects the predictions and help alignment with existing physiological mechanism. Building upon existing surveys on interpretable and strong AI for Electroencephalography applications^13^.

This article proposes an Explainable Neural Network-Driven Learning Model for Neurodegenerative Disorder Diagnosis (XNNLM-NDD). The core contributions of this work are listed as follows:

- Employ a hybrid feature selection method combining mRMR and ReliefF to select the most relevant and non-redundant features, which helps improve the learning capability of the model.
- Adopt a Feature Tokenizer-Transformer (FT-Transformer) model for capturing complex feature interactions in tabular biomedical data.
- Employ an improved optimization technique, namely Cycle-Norm-Adam (CN-Adam), which integrates a cyclic exponential decay learning rate with gradient norm constraints to accelerate convergence and improve the generalization through dynamic learning rate adjustment.
- Integrate SHAP-based explainability to provide a better understanding of feature contributions in the classification process.
- Conduct extensive experiments on benchmark Huntington Disease Dataset and demonstrate the supremacy of the proposed model under diverse performance measures such as Accuracy, Precision, Recall, F1-score and, MCC

## Related works

In this section, a brief review of related works on Huntington’s disease is presented along with identified research gaps. Ali^14^, introduce AI applications in analyzing multi-modal neuroimaging data, comprising magnetic resonance imaging (MRI), electroencephalography (EEG) and positron emission tomography (PET) to improve accuracy of diagnostic. By leveraging convolutional neural networks (CNNs) and transformer-driven techniques, AI models could recognize subtle structural and functional brain abnormalities suggestive of neurodegeneration before clinical symptoms manifest. In addition, ML algorithms track disease progression, predict cognitive impairment trajectories, and stratify patients according to personalized risk factors through processing longitudinal patient data. Dash et al.^15^ introduce a meta-analysis to recognize DEGs utilizing three Gene Expression Omnibus (GEO) microarray datasets on various platforms relation with HD-affected brain tissue, using both strict and relaxed criteria to recognize various expressed genes. Then, concentrate only on genes recognized by the inclusive threshold, author utilized 19 type of ML methods to discover the common genes that contributed to the top three chosen ML system and the shared genes, which had an effect on the ML technique and were observed in the meta-analysis utilizing the stringent setting has been chosen. Saravanan^16^ illustrates the DeepRepeatHD, a DL architecture which, integrates 1D-CNNs and transformers to process raw nucleotide sequences when integrating multi-omics information’s.

Yamini et al.^17^ inspected the genetic mechanisms for the HD conditions and assessed how these stages contribute to HD and epilepsy identification and development prediction with respect to the effectiveness of prescribed quantities by employing the various data mining methods. Dash et al.^15^ implemented bioinformatics and an ML model to make a strong architecture that combined a distinctively expressed gene (DEG) medical evaluation with ML to choose the possible biomarkers. To recognize robust DEGs in HD-impacted brain tissue, we combined three GEO microarray studies utilizing different platforms via a meta-analysis approach. In^18^, a ranking-based ensemble method that depends on the weighted approach of DL methods is developed. The entire modeling process of this developed technique combines three stages. During the first stage, the preprocessing methods have been utilized to clear the noise in databases to make them consistent with respect to DL methods, as it mainly affects their effective outcome.

Felix et al.^19^ presented efficient, rapid, alternative, and lower-cost solutions that can be assisted by ML approaches in which the analysis and difference of neurodegenerative disorders (NDDs), namely amyotrophic lateral sclerosis, parkinson’s disease (PD), and HD. Such diseases are attributed to the advanced loss of neurons, with no cure, and analysis is mainly based on medical data. With the help of innovative features removed from gait signals by employing the dynamic, various, and harmonic fluctuation. Ghofrani-Jahromi et al.^20^ implemented the stratifications of HD persons for medical experiences. To apply comprehensive brain parcellation to longitudinal MRI data and quantify lateral ventricular expansion over three years, which can be employed as the target variables for the diagnostic RF methods. The techniques have been trained under different integration of features, such as brain image-based features, cognitive and motor valuation score biomarkers, and genetic load. Schwartz et al.^21^ implemented an innovative gait detection method for persons with movement disorders, such as HD, in day-to-day life. The J-Net model, inspired by U-Net, uses pre-trained self-supervised learning and accelerometer data to classify gait patterns in HD, PD, and healthy individuals, and its performance is evaluated using both in-lab and real-life data.

Gao et al.^22^inspected the fundamental mechanisms of HD pathology by applying the single-cell sequential database. This method is employed by using single-cell RNA sequencing to find various patterns of gene expression amongst HD and healthy cells. Sanchez-DelaCruz et. al^23^. developed the ML technique efficiency to categorize the PD and HD that relies on the biomarker data acquired non-invasively using healthy controls and individuals. Additionally, the datasets comprising biomarker information by using the sensors that have been gathered from PD, HD persons, and healthy persons. An automated selection technique is utilized for classifying the diseases through the exceptional Mexican datasets of human gait biomarkers. The existing models for HD classification, including methods, objectives, datasets, and key findings, are given in Table 1.

**Table 1:** Related works on the HD detection system.

Authors	Years	Methods	Objectives	Dataset	Key findings
Ali^14^	2022	CNNs, transformer models,	AI applications for early detection, cognitive-decline prediction, and brain-imaging biomarker identification in neurodegenerative diseases	MRI, PET, EEG	AI (ML/DL) can detect subtle structural and functional brain abnormalities
Dash et al.^15^	2025	ML techniques	Identify novel biomarkers for Huntington’s Disease via differential expression meta-analysis	gene-expression datasets	Reported novel candidate gene biomarkers and improved discriminatory performance for Huntington’s Disease classification using ML
Saravanan^16^	2025	Hybrid CNN–Transformer framework	To Improve Huntington’s Disease severity prediction beyond Random Forests by using a hybrid CNN-Transformer	Multi-omics datasets	Hybrid CNN-Transformer outperformed Random Forest baselines on severity prediction tasks using multi-omics
Yamini et al.^17^	2026	SVM, RF, and gradient boost approaches	To improve the diagnostic accuracy of neurogenetic diseases using data mining techniques	KEGG and UniProt datasets	Accuracy of 94.7%
Dash et al.^15^	2025	ML models	To analyze differentially expressed genes to improve the early diagnosis of HD	GSE3790_ GPL96, GSE3790_ GPL97, and GSE26927 datasets	AUC of 95%
Goyal et al.^18^	2025	DL methods	To compare binary and multiclass classification performance for AD and PD diagnosis	ADNI and PPMI datasets	accuracy of 97.18% and 97.85%
Felix et al.^19^	2024	ML and DL techniques	To reduce diagnostic time and patient effort through optimized gait signal analysis	PD dataset	Specificity and sensitivity range from 96 to 100%
Ghofrani-Jahromi et al.^20^	2024	XAI and ML models	To enhance the prediction of disease progression through ventricular enlargement analysis	PREDICT, TRACK, TrackON, and IMAGE datasets	Accuracy of 81%
Schwartz et al.^21^	2024	U-Net and J-Net methods	To leverage self-supervised learning for improved activity recognition in NDDs	PD dataset	AUC-ROC of 0.92
Gao et al.^22^	2024	ResNet algorithms	To discover biologically relevant genes influencing HD pathology	iPSC-derived neurons, astrocytes, and neurons datasets	F1 score of 96.53%
Sanchez-DelaCruz et al.^23^	2024	ML techniques	To implement an automated model selection approach for NDD	PD dataset	Precision of 0.893%

## Research gaps

Based on the existing literature, numerous limitations can be observed:

- Yamini et al.^17^ used numerous feature selection technique with ML methods for the identification of HD. Although the technique depends on standard FS techniques, it cannot efficiently handle feature redundancy or intricate feature interactions corresponding to the data.
- Goyal et al.^18^ presented a ranking-based ensemble DL method for classifying neurodegenerative disorders, even though this technique’s prediction outcome improves computational complexity, it lacks a structured mechanism for learning feature-level correlation in tabular data.
- Ghofrani-Jahromi et al.^20^ implemented an ML method with imaging and medical features for risk stratification. Though the model primarily highlights the prediction of disease development, it cannot utilize an innovative DL model for correct classification.
- Gao et al.^22^ used the DL method with SHAP for evaluating the gene expression database in HD. Whereas the model offers explainability, it does not overcome FS or optimization complexity, and is also restricted to particular data types.

Hence, there is a necessity for an effective and explainable model to deal with feature redundancy and gain complex correlations in medical data. Thus, the proposed XNNLM-NDD technique has developed an efficient and explainable model to improve the accuracy and consistency of diagnosing neurodegenerative disorders.

## Proposed methodology

**Fig. 1:**
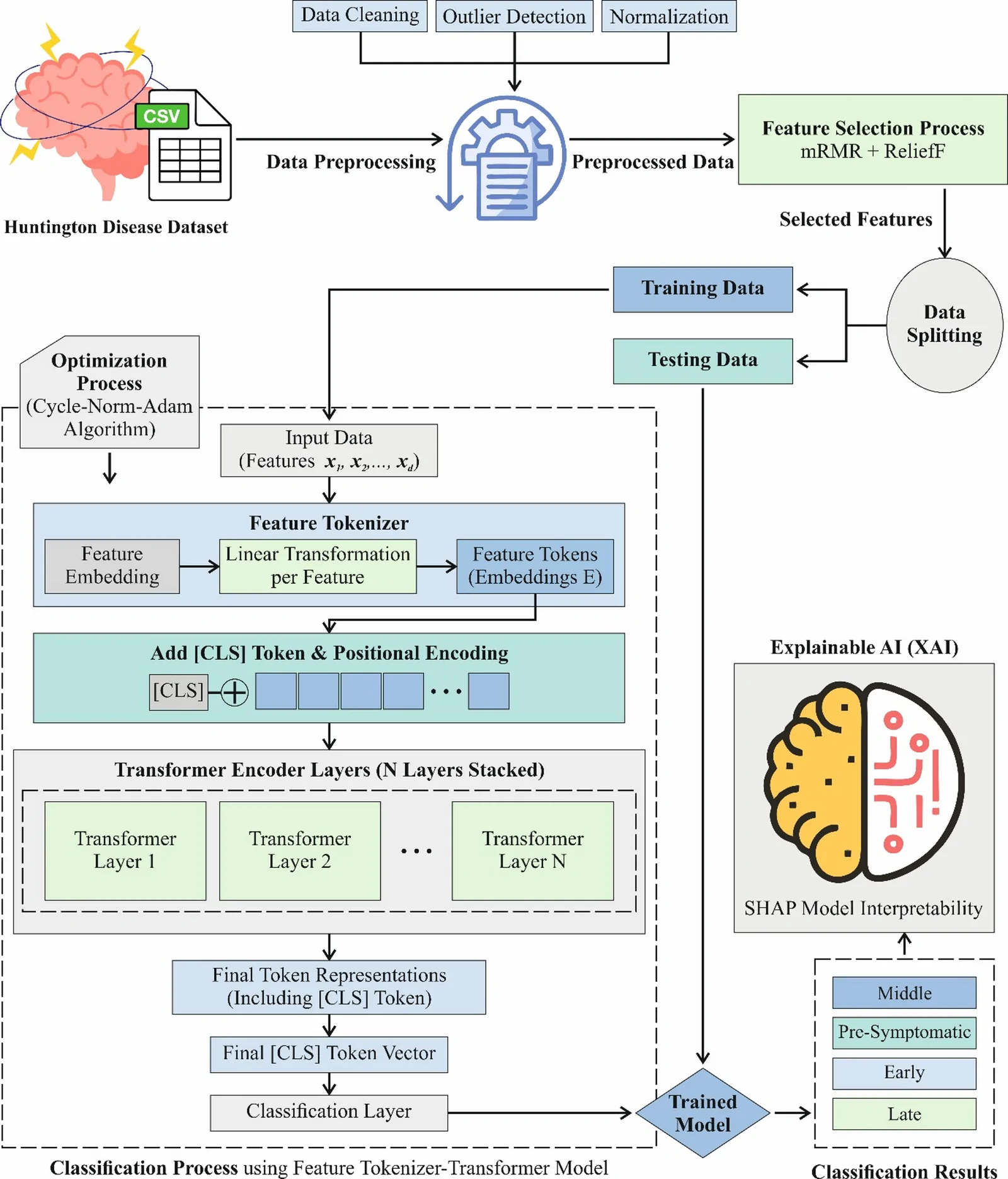
Workflow of the proposed XNNLM-NDD framework.

This section presents the overall pipeline of the proposed XNNLM-NDD model, which includes preprocessing, hybrid feature selection, classification, optimization, and explainability. The overall workflow of the proposed XNNLM-NDD framework is displayed in Fig. 1. The model consists of preprocessing, feature selection, classification, optimization, and explainability stages. First, the input Huntington’s disease dataset is preprocessed by cleaning, detecting outliers using the IQR method, and applying Z-score normalization to make the data suitable for training. After preprocessing, the cleaned data is passed through a hybrid feature selection process using mRMR and ReliefF for choosing the most relevant attributes and reducing redundancy. The selected features are then provided to the FT-Transformer model for classification, which assists in learning complex relationships between clinical attributes. The model is trained using the CN-Adam optimizer to improve convergence and performance. Finally, SHAP is employed to explain the model predictions and comprehend the contribution of each feature towards the final output.

### Data preprocessing

Preprocessing is a significant phase in biomedical data analysis, as a raw medical database might cover missing values, noise, and unreliable feature distributions. So, accurate pre-processing is essential for enhancing the quality of data and ensuring improved performance of the method.

*Data Cleaning*: The database is cleaned by removing duplicate samples or null values^24^. The samples that comprise any inaccessible values are deemed rectifiable. Moreover, data cleaning is intended to eliminate the insignificant features by ensuring they are in line with the feature distribution analysis. The features that are not associated with the outcome features are eliminated. The expression is represented in Eq. (1).

1 $$f\left(x\right)\leftarrow {d}_{i}=0,{x}_{i}\le 0$$

whereas the input value ${x}_{i}$ is less than or equal to zero, then the corresponding output/decision variable ${d}_{i}$ is set to 0.

*Outlier Detection Using the IQR Method*: In any given database, some values are very out of range compared with the maximum samples, which might mislead the model in training. Then, outlier removal is accomplished utilizing the Inter‐Quartile Range (IQR) approach.

2 $$IQR={q}_{3}-{q}_{1}$$

The IQR value is attained by calculating the variance among the 3rd and 1st quartiles, and values outside this range were classified and capped. This procedure aids in minimizing the impact of extreme values and assures a steadier data distribution.

*Normalization*: Normalization is applied for scaling the feature values into a standard range, which supports enhancing the learning model convergence. In this context, the Z-score normalization is used to convert the data to a distribution with a 0 mean and unit variance. The z-score normalization is computed as:

3 $$z = (x - \mu ) / \sigma$$

In this Eq. (3), *x* signifies individual data point, μ implies average, and σ denotes the standard deviation. This procedure assures that each feature contributed similarly in model training and enhances the steadiness of the classifier method.

### Hybrid feature selection strategy

To enhance the quality of predictive learning, an efficient feature selection mechanism is integrated after preprocessing. The hybrid FS method has been utilized by combining the ReliefF and mRMR techniques to efficiently recognize the useful features for HD classification.

#### The mRMR algorithm

The mRMR intends to choose the most important features that are the independent ones^25^. Then, the features with higher correlations with the class must be selected; these features can be defined by the relevance measures, and features with lower correlation amongst themselves have been defined by the redundancy calculations. By employing the determined measures, the weights of every individual feature can be measured for the class description in the HD dataset.

4 $$w= \mathrm{m}\mathrm{a}\mathrm{x} \left(\frac{1}{\left|S\right|}{\sum}_{{s}_{i}\epsilon s}I\left({s}_{{i}{^\prime}}d\right)-\frac{1}{|S{|}^{2}}{\sum}_{{f}_{i},{f}_{i}}I\left({s}_{{i}{\prime}}{s}_{j}\right)\right)$$

where *w* is a final computed importance value. And ${s}_{i}$ is a data instance, *d* is a decision value or class information.

#### The ReliefF algorithm

The ReliefF method depends on the evaluation of feature neighborhoods. Its core exists in enabling features in adjacent classes related to the similar class and discriminating compared to alternative classes. It enables us to find a measure of the relevance of every feature for class description. The relevance calculation $\mathcal{W}f$ for the feature in the ReliefF technique has been determined for annotations gathered in an *n*‐element training phase. To get every element *x* in this phase, the parameter *k* is the number of the closest neighbors of the same class, named as hits *h*, and the method *k* nearest neighbors (K-NN) of alternative categorization, termed as misses *m*. Later, for every individual feature, the weight rises through the distance at the point *x* to *m* and reduces via the distance within *x* to *h* for classifying the efficient diseases.

5 $${w}_{f}\left(m, k\right)=\frac{1}{nk}{\sum}_{j=1}^{n} \left({\sum}_{j=1}^{k}|{x}_{i},{m}_{ij}|-{\sum}_{j=1}^{k}|{x}_{i},{h}_{ij}|\right)$$

where the *t* term has been chosen, the medical features must be represented through a chosen number of features, and the final training phase must establish the increased feature subsets gained from the hybrid selection method. In order to get the datasets, the features have been decided based on their relevance, mainly to reduce their significant values. Thus, only the highest-ranked features must be included in the final feature sets for classifications.

### Deep learning-based classification model

**Fig. 2:**
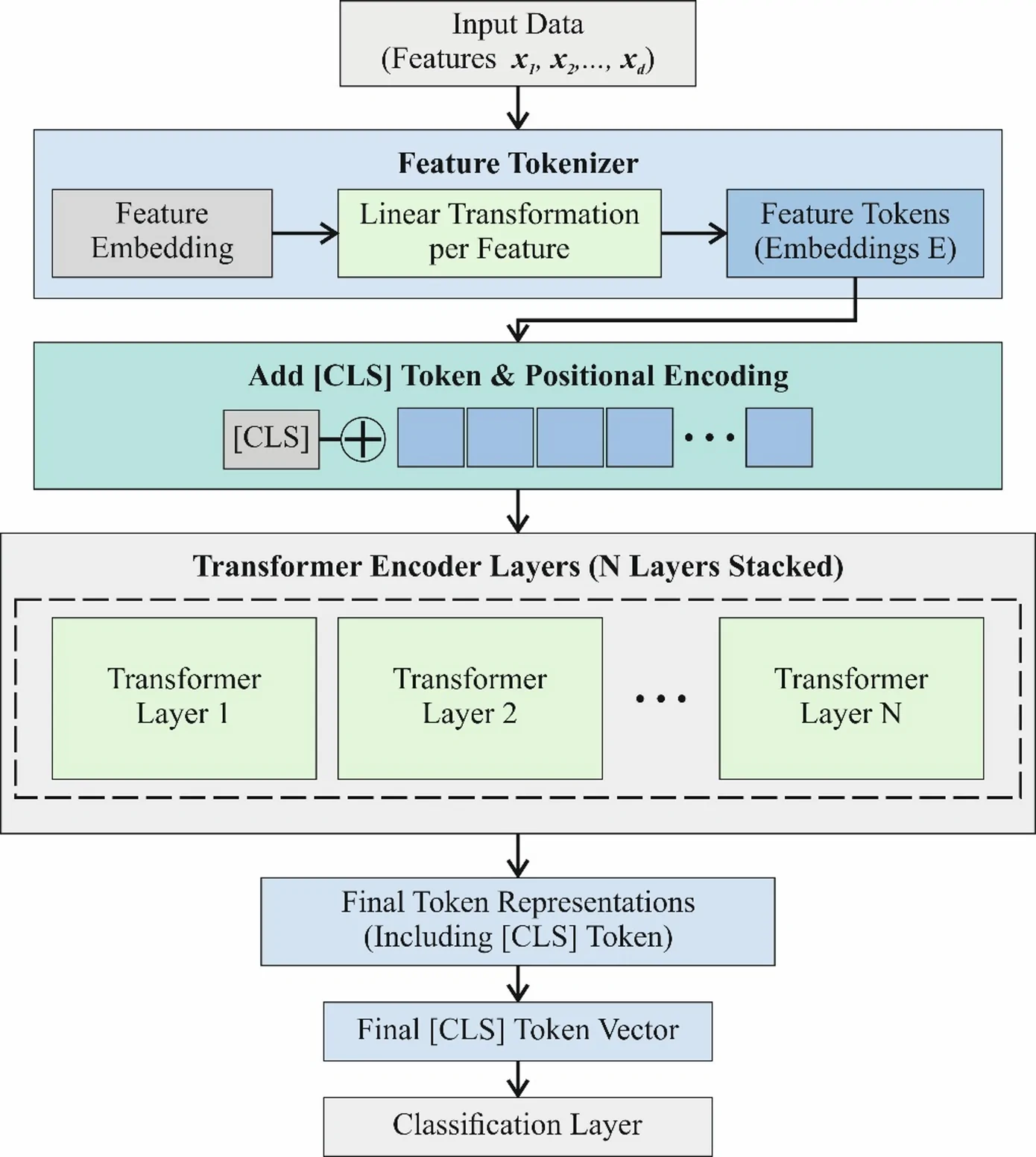
Overall FT-Transformer architecture.

The optimally selected features are then employed for DL-based classification of HD disease depending on the chosen features. The FT-Transformer is a modified version of the transformer architecture designed to overcome the vanishing gradient problem faced by RNNs in handling long sequences^26^. To tackle these issues by introducing the idea of attention in an encoding and decoding setting. Fundamentally, both the encoding and the decoding were transformer layers. At the same time, conventional neural network techniques frequently face complexity in taking into account intricate interactions among the medical characteristics, particularly in biomedical data, where feature relationships are not simple. Currently, the Transformer architecture has demonstrated its greatest impact in image recognition, machine translation, and text prediction. That is the aim of the proposed XNNLM-NDD method, which applies an FT to efficiently manage structured biomedical datasets, thus concentrating on the efficiency with tabular data. Rather than utilizing an encoding and decoding transformer, they utilize the FT and numerous layers of a single transformer. It supports the method for better learning feature dependency and enhances the representation of clinical attributes for disease detection. Figure 2 shows the overall FT-Transformer architecture.

The FT element of the FT‐Transformer executes the feature data extractor. The FT alters input to embedded and is intended to manage both categorical and numerical attributes concurrently. Meanwhile, the HD database contains continuous clinical attributes; the embedded attributes were represented as follows:

6 $${T}_{j}={b}_{j}+{f}_{j} \in {\mathbb{R}}^{d} {f}_{j}={X}_{j}\to {\mathbb{R}}^{d}$$

The model converts input biomedical data ${X}_{j}$ into a learned feature representation ${f}_{j}$, integrates it with a bias term ${b}_{j}$, and produces a transformed feature vector ${T}_{j}$ for further classification or diagnosis tasks. Hereafter, the classification (CLS) token was inserted to ultimately utilize the final representation for creating the predictive model.

**Fig. 3:**
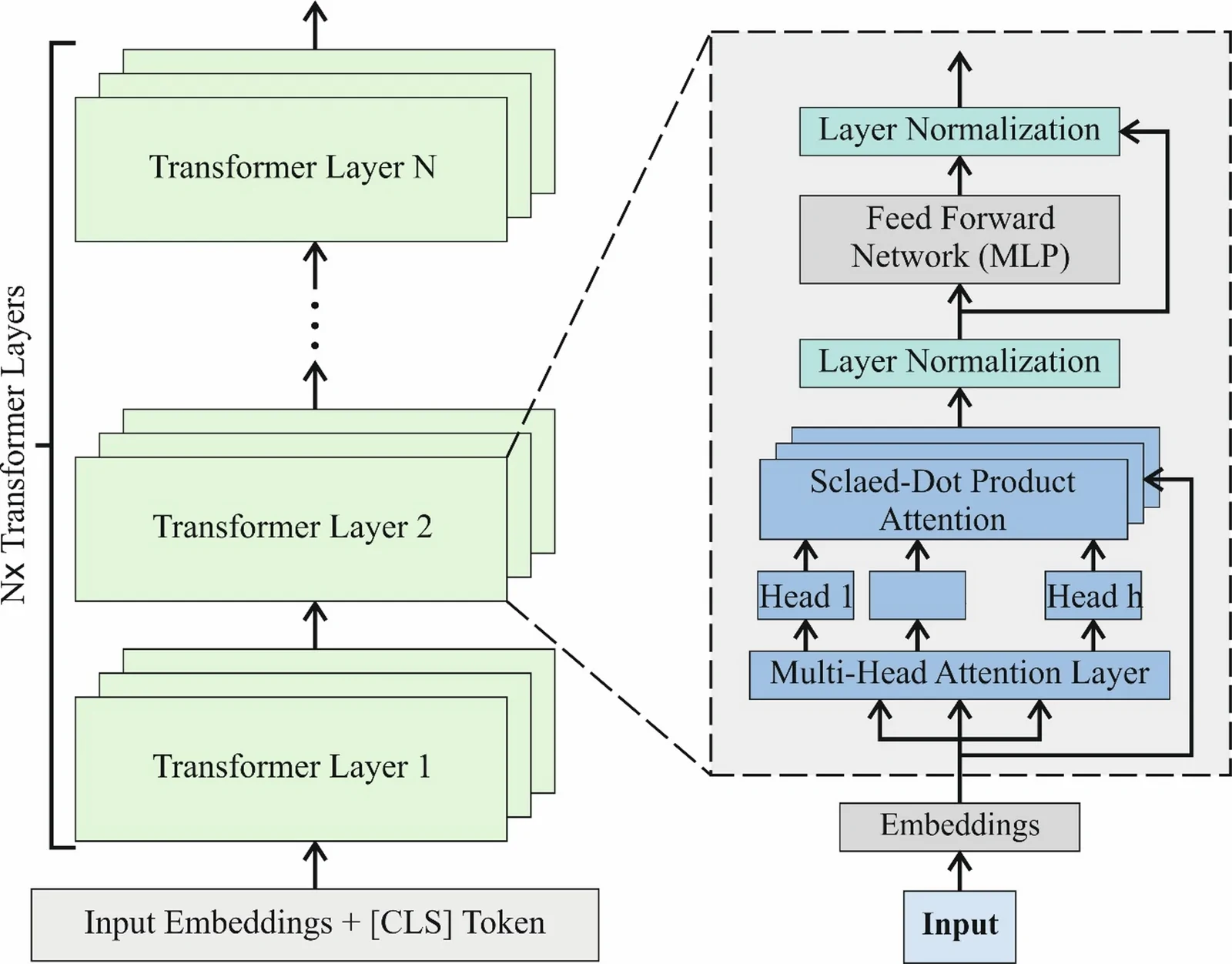
Internal architecture of the FT-Transformer.

Mutually, the embedded and the CLS token are passed to the stack of transformer layers. A single layer is equal to the encoding transformer. This section commences with a Multi‐Head Attention (MHA) module. MHA functions by executing multiple, independent attention mechanisms (AM) in parallel. The AM imitates cognitive attention when processing feature interaction. However, an AM contains 3 methods. Initially, a procedure reads the embedded and divides them into distribution vectors. Next, a list of characteristics was learned as a sequence of representations that could be recovered later. At last, the representation part that is required for the classification was used. This mechanism is valuable in classifying which medical features contribute further to the diagnosis and how they relate to one another. Next, in the MHA mechanism, the data is fed to the Feed Forward method. This is a multi-layer perceptron architecture utilizing Rectified Linear Unit (ReLU) nonlinearities, featuring a shortcut connection that bypasses the intermediate weight layers. These elements aid in steadying the training procedure and enable the method for learning deeper feature representations efficiently. Figure 3 demonstrates the internal architecture of the FT-Transformer showing transformer layers and attention mechanism.

### Model optimization using CN‐Adam

To further enhance convergence and classification effectiveness, the presented model is optimized using the CN-Adam optimizer. The Adam optimization method combines momentum and adaptive learning rate to train neural networks by adjusting the learning rate based on gradients and improving convergence speed^27^. The Adam method enhances the parameters by retaining 1st order and 2nd order moment estimations for all the parameters. While it updates the parameters, the Adam integrates the dual estimation and exceeds bias corrections to assure unbiased estimation. Through the utilization of exponential moving averages, the Adam is proficiently combined and updated prior to gradient data to give greater consideration to the optimization method of the parameters. At every individual iteration, the loss function gradient corresponding to the parameters can be measured by employing this mathematical form.

7 $${g}_{t}={\nabla}_{\theta }{f}_{t}\left({\theta}_{t}\right)$$

where the term ${g}_{t}$ represents the gradient at step *t*. The parameter vector ${\theta}_{t}$ can be updated with respect to time. The 1st and 2nd-order moment estimation in the Adam method shows the moving average of the 1st and 2nd orders of the gradient. Such calculations have been determined as represented in this mathematical form,

8 $${m}_{t}={\beta}_{1}{m}_{t-1}+\left(1-{\beta}_{1}\right){g}_{t}$$

9 $${v}_{t}={\beta}_{2}{v}_{t-1}+\left(1-{\beta}_{2}\right){g}_{t}^{2}$$

whereas the terms ${m}_{t}$ and ${v}_{t}$ are parameters used in the Adam Optimizer. The Adam optimizer technique presents a bias correction method to overcome possible bias in the 1st and 2nd order moment assessments while the decay rate is lower in the preliminary phase. The mathematical form of the bias correction can be denoted as given below,

10 $${\widehat{m}}_{t}=\frac{{m}_{t}}{1-{\beta}_{1}^{t}}$$

11 $${\widehat{V}}_{t}=\frac{{v}_{t}}{1-{\beta}_{2}^{t}}$$

The parameter update *θ* is calculated, and then all the iterations can be determined in this mathematical form.

12 $${\theta}_{t+1}={\theta}_{t}-\frac{lr}{\sqrt{{v}_{t}}+\varepsilon }\cdot {\widehat{m}}_{t}$$

Here, the mathematical form *lr* refers to the learning rate, and ε indicates a smaller positive number to prevent a 0 denominator. The main limitations of the Adam optimization are the sensitivity parameter to the (ALR). Thus, while utilizing the Adam optimization, attention has been required as a reward for its sensitivity to the gradient and learning rate, and then the unpredictability and degradations of the convergence efficiency that can occur in particular conditions. The cyclical learning rate (CLR) is a technique for enhancing the training method of NNs. This method presents the concept of regularly correcting the learning rate. In contrast to standard learning rate scheduling techniques, the CLR permits the learning rate to be modified at the time of the training method. Especially, it can be changed to a learning rate method and add higher flexibility for the model training phase. At the training phase, the CLR impacts the learning rate to modify within the predetermined ranges, permitting the model to work with various data distributions and enhancing the generalization capability. The learning rate is updated using a cyclic exponential decay strategy, where the rate varies periodically within a defined range and gradually decays over iterations.

13 $$c = \left\lfloor {1 + 2Tt} \right\rfloor$$

14 $$X=\mid \frac{t}{T}-2c+1\mid$$

15 $$\eta ={\eta}_{min}+({\eta}_{max}-{\eta}_{min})\cdot \mathrm{m}\mathrm{a}\mathrm{x}(\mathrm{0,1}-X)$$

16 $${\eta}_{t}=\eta \cdot {\gamma }^{t}$$

### Explainability with SHAP

To improve transparency and clinical interpretability, explainability analysis is performed by SHAP. Furthermore, Explainability is a significant part of improving model transparency by classifying and emphasizing the most influential features that contribute to the predictive method. By using SHAP, which is based on cooperative game theory and allocates an importance score for all features corresponding to a prediction^28^. With the coalitional game with *N* players, the Shapley value ${\phi}_{i}$ for a player *i* is represented as:

17 $${\phi}_{i}= {\sum}_{S\underset{\_}{\subseteq }N\setminus \left\{i\right\}} \frac{\left|S\right|!\left(\left|N\right|-\left|S\right|-1\right)!}{\left|N\right|!}\left[v\left(S\cup \left\{i\right\}\right)-v\left(S\right)\right]$$

Here, *S* implies a subset of features excluding feature *i*, and *v(S)* indicates the model output for that subset. The difference shows how much feature *i* contributes to the prediction. SHAP explains complex model outputs by approximating them with a simpler linear model based on selected feature combinations.

18 $$g\left({x}^{^{\prime}}\right)={\phi}_{0}+{\sum}_{i=1}^{M}{\phi}_{i}{x}_{i }^{^{\prime}}$$

In this Eq. (18), ${\phi}_{0}$ represents the baseline outcome and ${\phi}_{i}$ the feature contribution *i*. Here, ${x}_{i}^{^{\prime}}=1$ implies that the *i*th feature was involved, and ${x}_{i}^{^{\prime}}=0$ otherwise. The Shapley value for variable *i* corresponds to a sample *x,* which is calculated as:

19 $${\phi}_{i}\left(f,x\right)={\sum}_{{z}^{^{\prime}}\underset{\_}{\subseteq }\mathrm{x}^{\prime}}\frac{\left|{z}^{^{\prime}}\right|!\left(M-\left|{z}^{^{\prime}}\right|-1\right)!}{M!}\left[{f}_{x}\left({z}^{^{\prime}}\right)-{f}_{x}\left({z}^{^{\prime}}\setminus i\right)\right]$$

Let ${z}^{^{\prime}}$ denote the simplified input subset ${x}^{^{\prime}}$, and ${f}_{x}({z}^{^{\prime}})$ with respect to the method outcome limited to those attributes. To analyze the features as per the absolute Shapley values, the contribution of every feature is interpreted for HD detection. Practically, the process offered insights into method decision-making and enhanced interpretability, while also safeguarding transparency. Experimental assessment also showed that the SHAP-driven explanation efficiently emphasized the most influential attributes that contribute to the predictive methods.

## Experimental results and discussion

In this section, a comprehensive analysis of the proposed method is presented, including dataset description, performance results, comparative analysis, and XAI results.

### Dataset description

The performance evaluation of the XNNLM-NDD technique can be inspected by utilizing Huntington Disease dataset^29^. This dataset gives organized data relevant to HD, an advanced neuro-degenerative disorder affects by the genetic mutations especially in the HTT gene. This can be comprised demographic, genetic, and medical data, thereby creating it useful for medical research, the ML applications, and prediction modelling. The overall numbers of 20 features can be primarily extracted, out of which 15 more relevant features have been chosen for additional evaluation after that the FS methods. Table 2 represents the dataset that comprises 4 classes namely middle, pre-symptomatic, early, and late phase with the overall samples are represented as 48,536.

**Table 2:** Details of dataset.

Classes	No. of Instances
Middle	12,289
Pre-Symptomatic	12,191
Early	12,032
Late	12,024
Total	48,536

### Explainability results

**Fig. 4:**
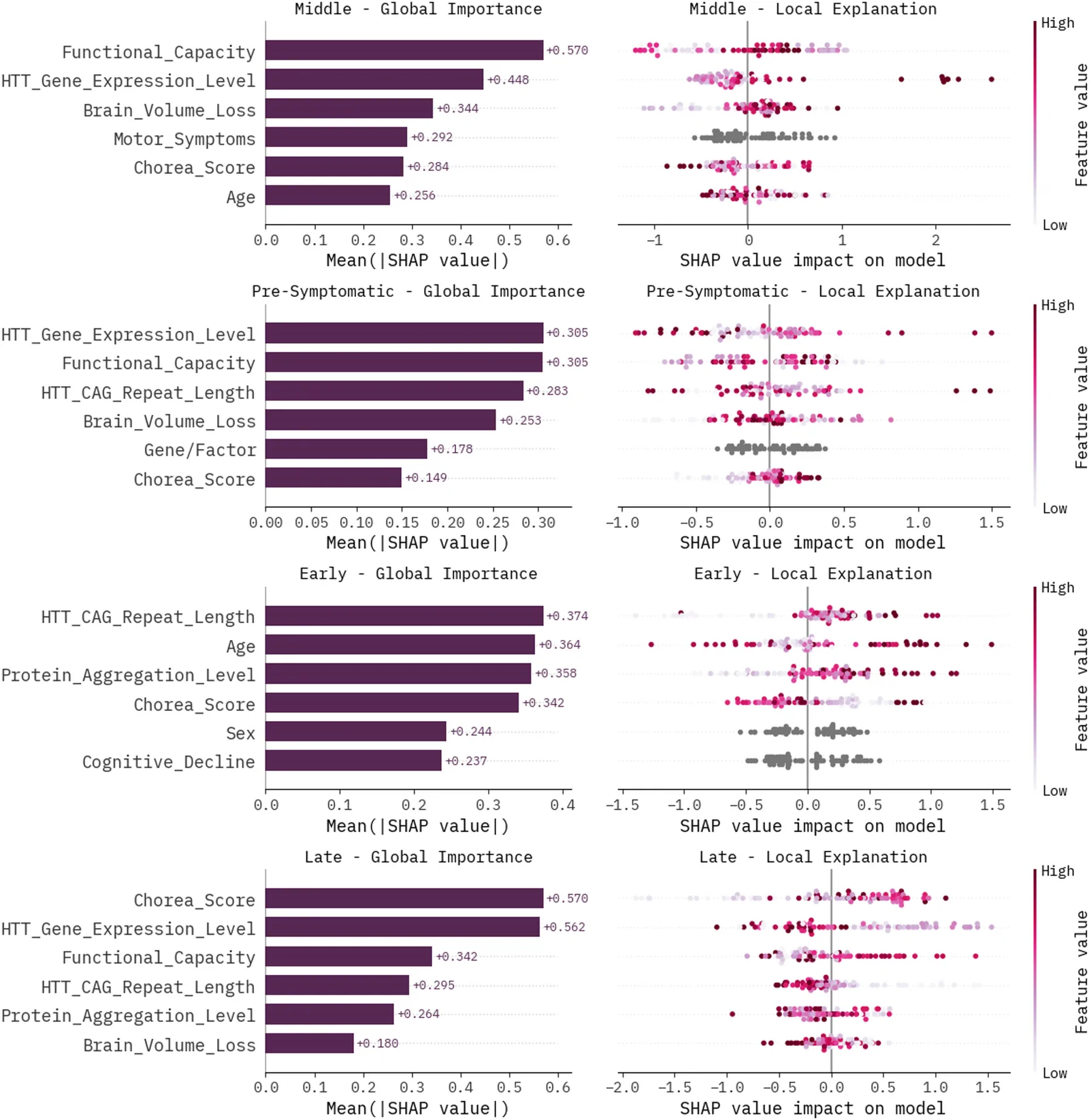
SHAP value for global and local explanations.

Figure 4 represents the SHAP-based global and local explanations for the proposed methods across four disease phases namely middle, pre-symptomatic, early, and late. The global importance exhibits the more significant features that offer to every individual class prediction, where brain volume loss, HTT Gene Expression Level, functional capacity, Chorea Score, and HTT CAG repeat length reliably emerge as main predictors. The local explanation demonstrates the distribution of SHAP values for individual instances, thus emphasising the feature impacts model output like positively or negatively. The color gradient denotes magnitude of feature values, where greater values can be normally contributed more stronger to the prediction. In the whole phases, the clinical and genetic conditions direct model decision-making, thus ensuring their relevance of strong predictive.

### Performance results

**Fig. 5:**
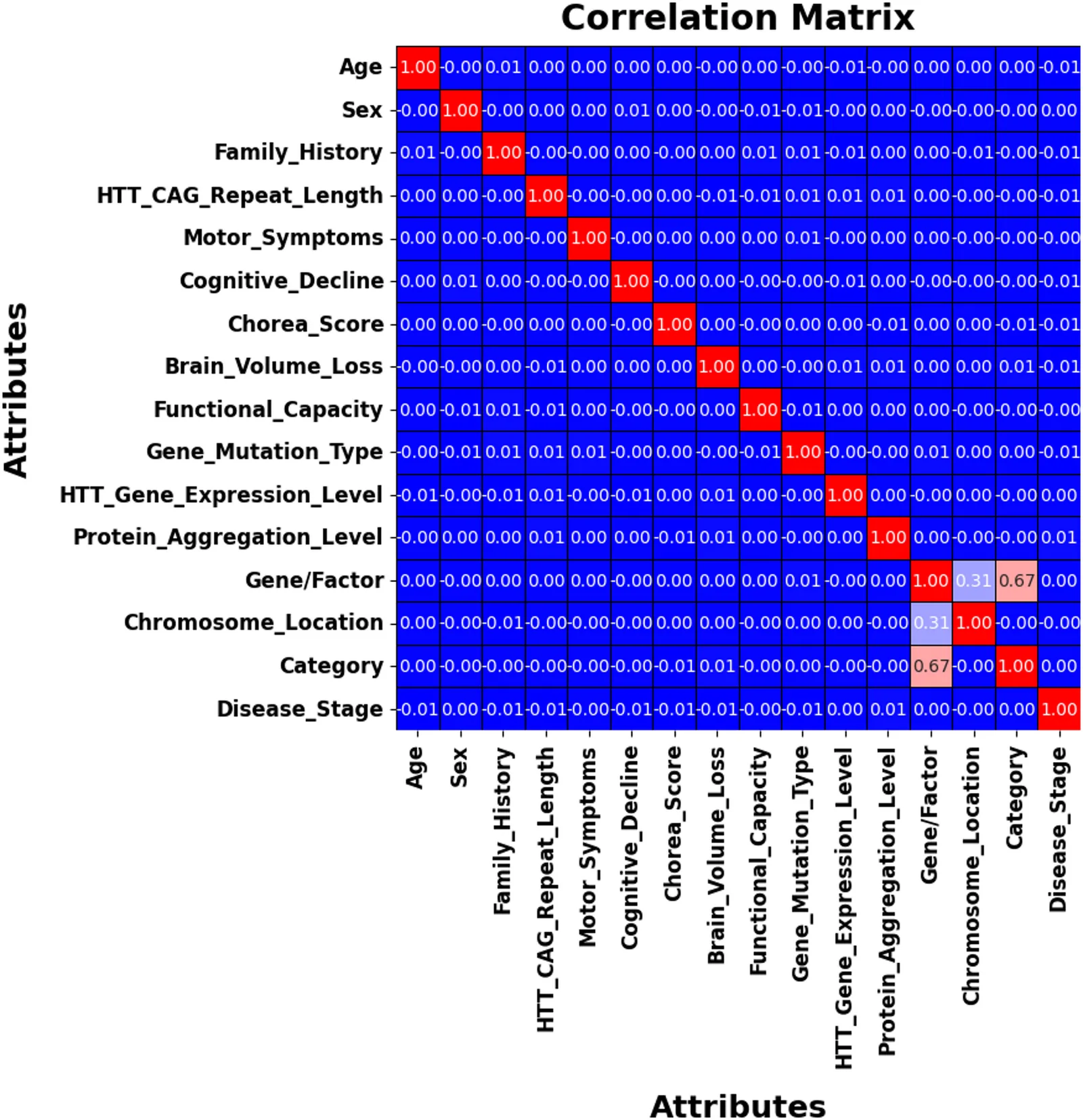
Correlation matrix of the XNNLM-NDD approach.

Figure 5 shows the correlation matrix of the proposed XNNLM-NDD system is accomplished correlation among demographic, genetic, and clinical features are employed in the dataset. Maximum features are demonstrated as extremely lesser or near-zero relationship, thus denoting lower redundancy and higher feature independence. But a few variables are such as Gene or Factor and Category exhibit reasonable correlation, thus offering their possible relevance in stages of disease prediction.

Table 3 show the HD classifier outcomes created by the XNNLM-NDD method using 80:20 of TRAP/TESP. The confusion matrices exhibit that the XNNLM-NDD technique successfully classifies all classes with higher accuracy. Furthermore, the HD classification results with 80% TRAP, the XNNLM-NDD system obtains average outcomes with $acc{ur}_{y}$ of 94.15%, $prec{i}_{n}$ of 88.47%, $reca{l}_{l}$ of 88.29%, ${F1}_{score}$ of 88.32% and MCC of 84.47%, Meanwhile, based 20% TESP, the XNNLM-NDD approach gains average outcomes with $acc{ur}_{y}$ of 93.99%, $prec{i}_{n}$ of 88.11%, $reca{l}_{l}$ of 87.94%, ${F1}_{score}$ of 87.94% and MCC of 84.02%, respectively.

**Table 3:** HD classifier result of the XNNLM-NDD method with 80:20.

Class Labels	$Accu{r}_{y}$	$Prec{i}_{n}$	$Reca{l}_{l}$	$F{1}_{score}$	MCC	Confusion Matrices
Middle	95.89	89.43	94.96	92.11	89.41	
Pre-Symptomatic	91.69	82.19	85.54	83.83	78.27	
Early	93.65	88.74	85.31	86.99	82.82	
Late	95.39	93.53	87.34	90.33	87.40	
Average	94.15	88.47	88.29	88.32	84.47	
Middle	95.90	89.21	95.43	92.21	89.53	
Pre-Symptomatic	91.67	81.68	85.64	83.61	78.07	
Early	93.54	88.56	84.49	86.47	82.27	
Late	94.87	93.01	86.20	89.48	86.20	
Average	93.99	88.11	87.94	87.94	84.02	

**Fig. 6:**
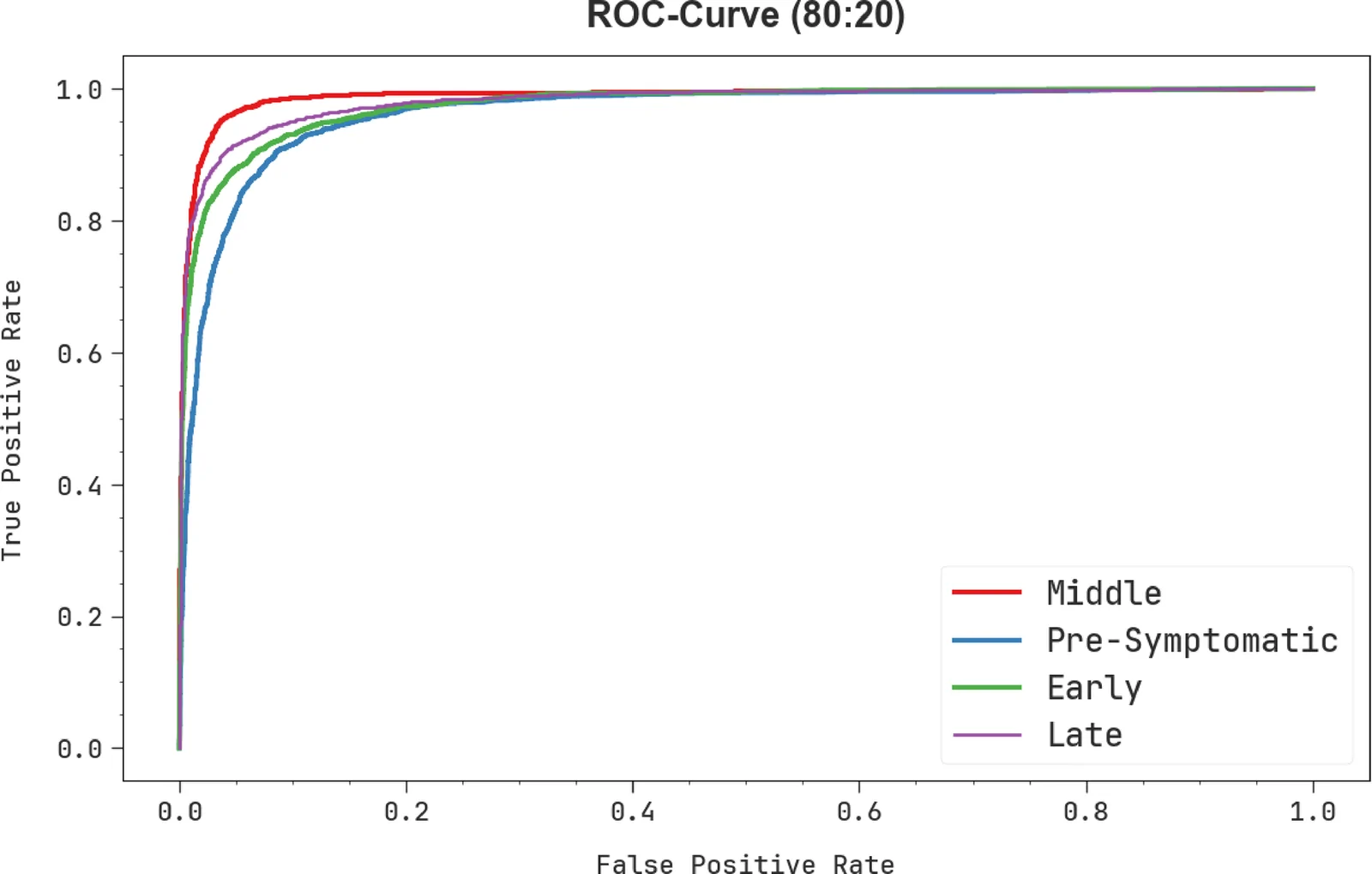
ROC-curve of the XNNLM-NDD method with 80:20.

Figure 6 demonstrate the ROC curve evaluation of the XNNLM-NDD system by using an 80:20 train-test split. The analysis shows that the model constantly obtains greater ROC scores for all classes, indicating efficient separation among categories. This persistent patten across classes emphasizes the stability of model and stronger in executing correct classification.

Table 4 portray the HD classifier outcomes created by the XNNLM-NDD method with 70:30 of TRAP/TESP. The confusion matrices reveal that the XNNLM-NDD system efficiently categorizes all classes with higher precision. Likewise, the HD classification outcomes at 70% TRAP, the XNNLM-NDD approach offers average outcomes with $acc{ur}_{y}$ of 96.48%, $prec{i}_{n}$ of 93.01%, $reca{l}_{l}$ of 92.97%, ${F1}_{score}$ of 92.96% and MCC of 90.64%, respectively. Also, based on 30% TESP, the XNNLM-NDD technique attains average outcomes with $acc{ur}_{y}$ of 96.50%, $prec{i}_{n}$ of 93.09%, $reca{l}_{l}$ of 93.00%, ${F1}_{score}$ of 93.01% and MCC of 90.71%.

**Table 4:** HD classifier result of the XNNLM-NDD method with 70:30.

Class Labels	$Accu{r}_{y}$	$Prec{i}_{n}$	$Reca{l}_{l}$	$F{1}_{score}$	MCC	Confusion Matrices
Middle	95.63	90.68	92.24	91.45	88.52	
Pre-Symptomatic	96.37	95.53	89.84	92.60	90.27	
Early	96.87	92.11	95.48	93.77	91.70	
Late	97.03	93.71	94.33	94.02	92.05	
Average	96.48	93.01	92.97	92.96	90.64	
Middle	95.57	90.29	92.42	91.35	88.38	
Pre-Symptomatic	96.42	95.54	89.71	92.54	90.26	
Early	96.90	92.14	95.78	93.92	91.87	
Late	97.14	94.38	94.10	94.24	92.34	
Average	96.50	93.09	93.00	93.01	90.71	

**Fig. 7:**
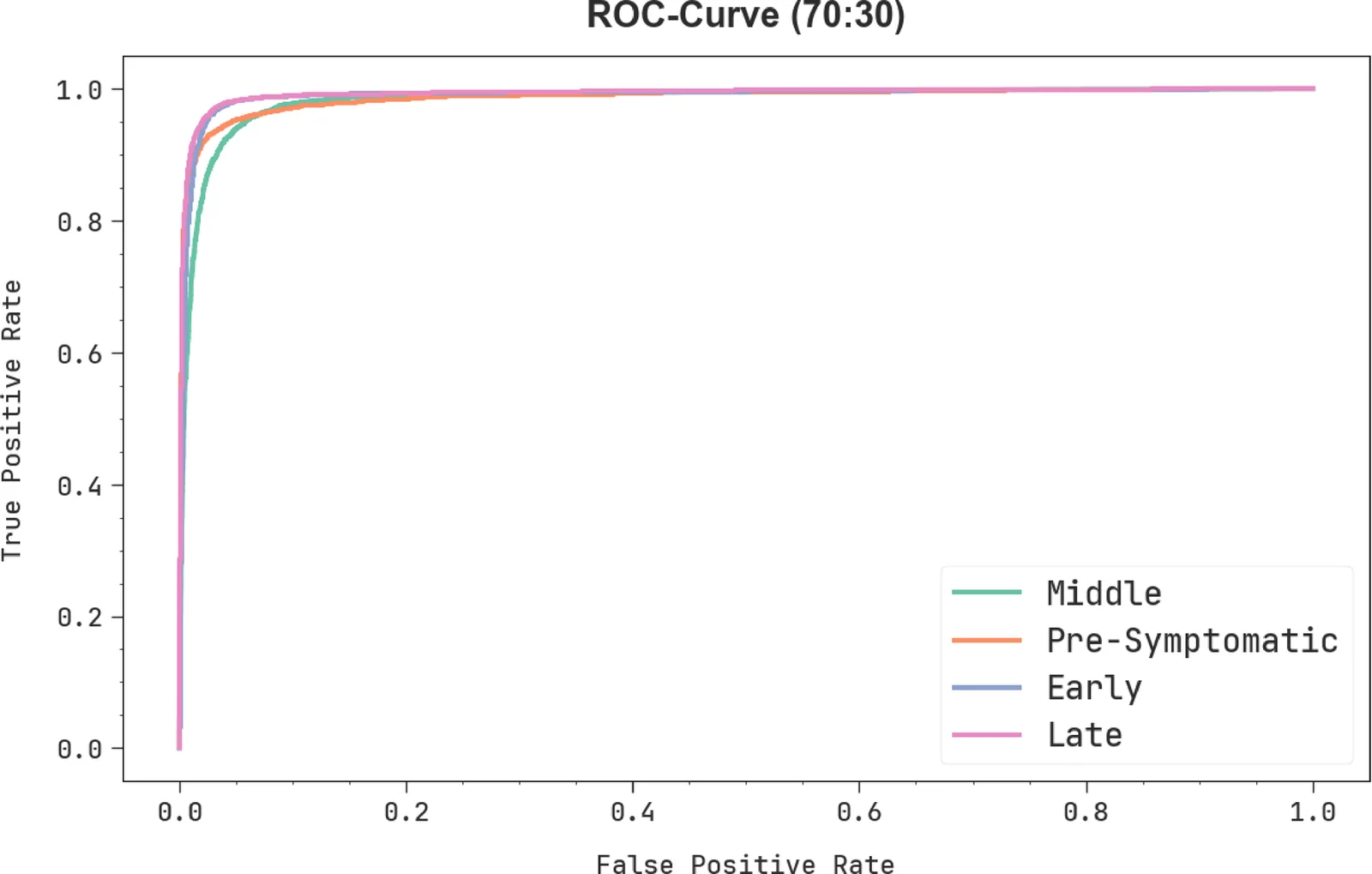
ROC-curve of the XNNLM-NDD method with 70:30.

Figure 7 exemplify the ROC curve evaluation of the XNNLM-NDD system employing the 70:30 train-test splits. The outcome evaluation indicates that the model constantly gains higher-level ROC scores for all classes, representing proficient separation among categories. This consistent form with classes highlights the steadiness of model and robustness to perform precise classification.

### Comparative analysis

The comparison evaluation of XNNLM-NDD approach with existing methodologies are represented in Table 5^30,31^. The experimentation outcome values are stated that the XNNLM-NDD method exceeded greater performances. Based on CT, the proposed XNNLM-NDD method achieves minimal value with CT of 1.06 s, while SVM, Logiboost, Ensemble Model, DCNN, Inception Time, PatchTST, and GRU approaches obtains maximal value with CT of 2.23 s, 7.89 s, 2.01 s, 7.27 s, 6.77 s, 6.56 s, and 7.06 s, respectively.

**Table 5:** Comparison result of the XNNLM-NDD method with other systems.

Models	$Accu{r}_{y}$	Error Analysis (%)	$Prec{i}_{n}$	$Reca{l}_{l}$	$F{1}_{score}$	Computational Time (sec)
SVM	81.80	18.20	80.23	84.64	82.08	2.23
Logiboost	94.40	5.60	90.23	91.44	90.93	7.89
Ensemble Model	55.30	44.70	61.57	59.65	62.08	2.01
DCNN	82.00	18.00	87.64	85.04	82.90	7.27
Inception Time	81.80	18.20	86.10	89.80	87.80	6.77
PatchTST	79.40	20.60	85.10	87.10	86.10	6.56
GRU	75.40	24.60	77.30	94.00	84.80	7.06
XNNLM-NDD	96.50	3.50	93.09	93.00	93.01	1.06

**Fig. 8:**
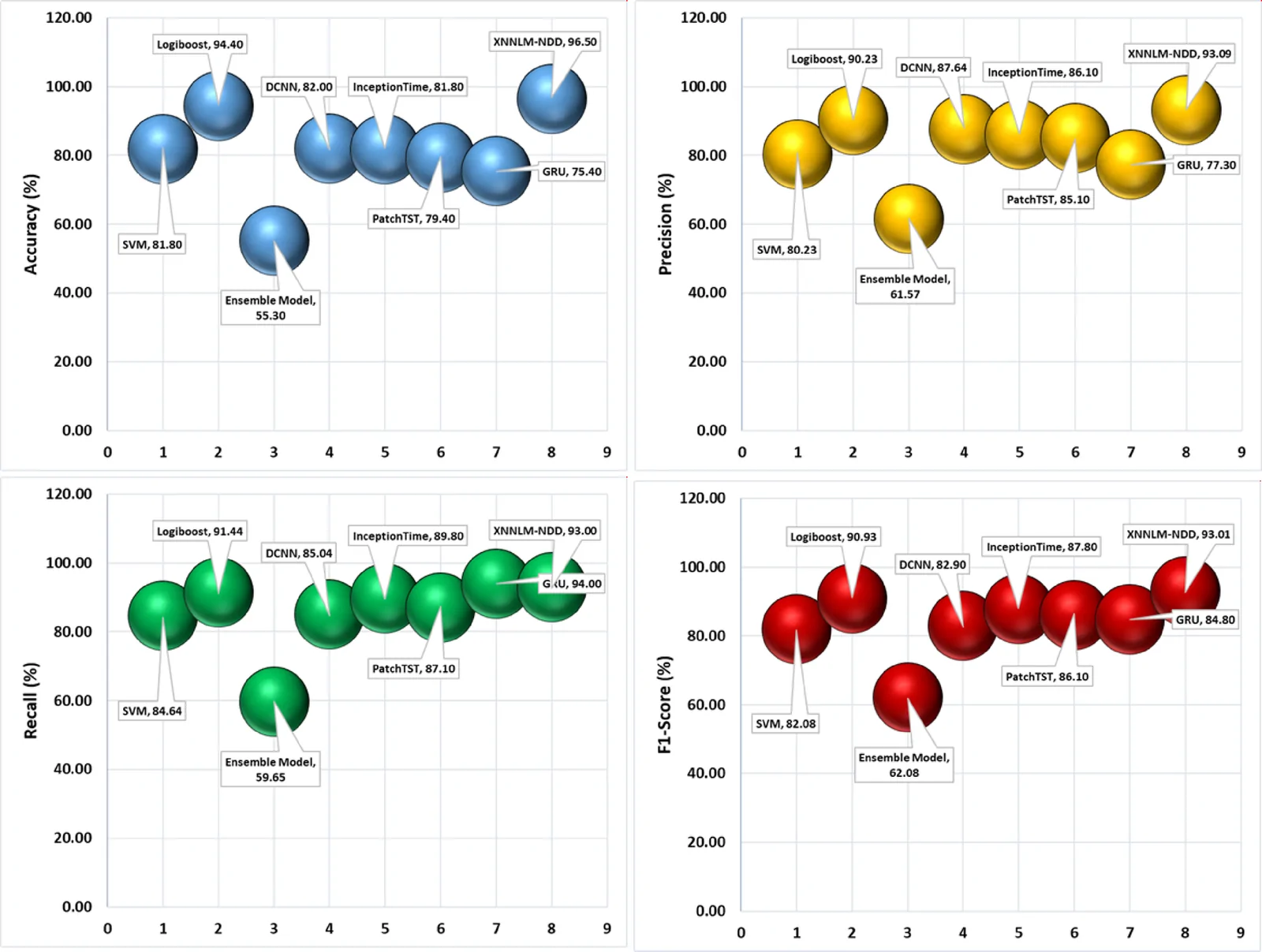
Comparative outcome of the XNNLM-NDD system with existing techniques.

Figure 8 show the comparison result of the XNNLM-NDD approach with other methodologies using metrices. The outcome implied that the Ensemble Model, PatchTST, and GRU approaches have obtained lower outcomes, whereas the SVM, DCNN, and Inception Time systems have attained slightly greater performance. In addition, the Logiboost model has gained reasonable outcome with $acc{ur}_{y}$ of 94.40%, $prec{i}_{n}$ of 90.23%, $reca{l}_{l}$ of 91.44%, and ${F1}_{score}$ of 82.08%. At the same time, the XNNLM-NDD approach has achieved higher performance with $acc{ur}_{y}$ of 96.50%, $prec{i}_{n}$ of 93.09%, $reca{l}_{l}$ of 93.00%, and ${F1}_{score}$ of 93.01%.

**Fig. 9:**
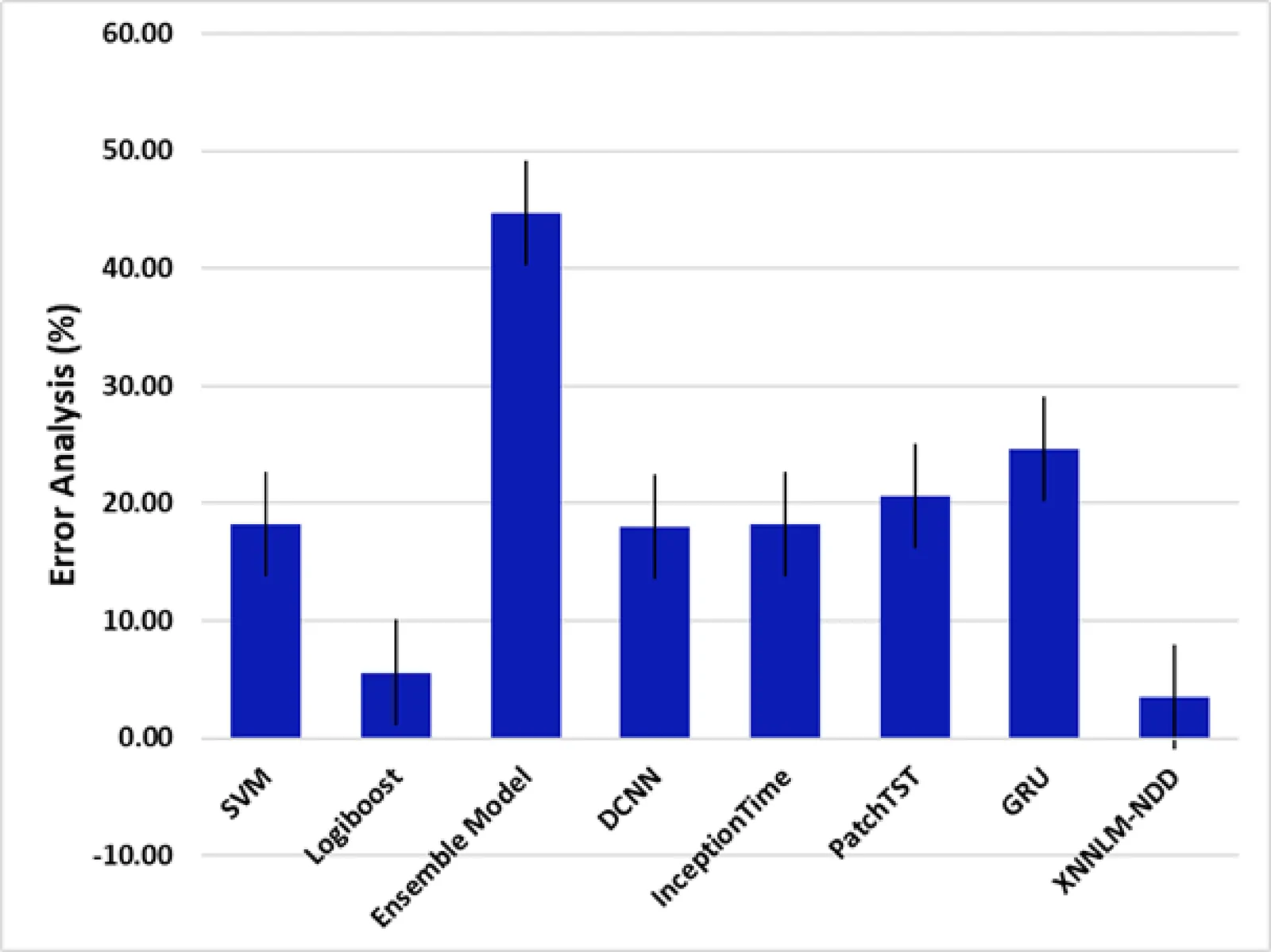
Error analysis of the XNNLM-NDD approach with other methods.

Figure 9 display the error analysis of the XNNLM-NDD method with existing techniques. The findings shown that the SVM, Logiboost, Ensemble Model, DCNN, Inception Time, PatchTST, and GRU methodologies have gained the highest outcome with error analysis of 18.20%, 5.60%, 44.70%, 18.00%, 18.20%, 20.60%, and 24.60%, while the XNNLM-NDD approach has reached the lowest outcome with error analysis of 3.50%, respectively.

An ablation study assesses the contribution of different constituents with a model by methodically eliminating or altering them to evaluate their influence under overall performance. This method supports to recognize the importance of every component and calculates that components provide numerous final outcomes. Through evolution of performance differences, research workers can improve the understanding of the model’s internal behavior. The useful insights are acquired and supported to improve and optimize the model for higher efficiency. Table 6 show the ablation study executed for the XNNLM-NDD approach. The result indicated that the mRMR Features only, ReliefF Features only, Hybrid Feature selection (mRMR + ReliefF), FT-Transformer (Transformer classifier), and FT-Transformer + hybrid feature selection (without CN-Adam optimization) methodologies have got inferior value with different metrics. For the meantime, the XNNLM-NDD (FT-Transformer + hybrid feature selection with CN-Adam optimization process) method has achieved superior value with $acc{ur}_{y}$ of 96.50%, $prec{i}_{n}$ of 93.09%, $reca{l}_{l}$ of 93.00%, and ${F1}_{score}$ of 93.01%

**Table 6:** Ablation study of the XNNLM-NDD approach with measures.

Models	$Accu{r}_{y}$	$Prec{i}_{n}$	$Reca{l}_{l}$	$F{1}_{score}$
mRMR Features only	93.24	89.94	89.89	89.81
ReliefF Features only	93.90	90.44	90.51	90.37
Hybrid Feature selection (mRMR + ReliefF)	94.55	91.14	91.21	91.10
FT-Transformer (Transformer classifier)	95.14	91.81	91.74	91.74
FT-Transformer + hybrid feature selection (without CN-Adam optimization)	95.94	92.36	92.27	92.51
XNNLM-NDD (FT-Transformer + hybrid feature selection with CN-Adam optimization process)	96.50	93.09	93.00	93.01

The results shown that the proposed XNNLM-NDD technique performs better than the existing approaches across all evaluation metrics, which specifies its efficiency for neurodegenerative disorder detection. The consistent performance observed in both training and testing phases demonstrates that the model generalizes well without overfitting. The ablation analysis also indicates that each component of the model contributes to enhancing the overall performance. In general, the combination of hybrid feature selection, transformer-based classification, and improved optimization is the main reason for the improved results. In addition, the SHAP-based XAI results offer clear insights into feature contributions, assisting to understand the model predictions and improving trust in the system.

## Conclusion and future work

In this manuscript, the novel XNNLM-NDD approach has been presented for analyzing clinical attributes and identifying disease patterns effectively for accurate diagnosis. The model performed feature selection using a hybrid combination of mRMR and ReliefF approaches to choose the most beneficial and non-redundant attributes from the dataset. For classification, the proposed model used an FT-Transformer method, which can capture complex feature interactions and enhance classification precision on structured medical data. Additionally, the model is enhanced using the CN-Adam algorithm. To ensure model interpretability, the SHAP-based XAI technique is leveraged to emphasize the contribution of each feature towards the final prediction. The experimental evaluation is conducted on the Huntington Disease Dataset, and the results demonstrate that the proposed XNNLM-NDD system achieved superior performance with an accuracy of 96.50% over recent models, signifying its effectiveness in progressive neurodegenerative disorder diagnosis. The proposed XNNLM-NDD model could be efficiently used in real-time clinical domains to help neurodegenerative disorder analysis and decision-making. Through incorporating the steps of preprocessing, hybrid feature selection, DL-based classification, and explainability analysis, the model could effectively process biomedical data and provide interpretable diagnostic estimation. Moreover, the integration of SHAP-based explainability allows medical experts to understand the contribution of individual clinical features, thus enhancing transparency, reliability, and practical applicability in monitoring Huntington’s disease progression and evaluating treatment results. However, the presented method is evaluated on a limited dataset, which may impact its generality to broader clinical scenarios. In future, the proposed work can be extended by using larger and more diverse datasets to further enhance the model performance. The model can also be examined on other neurodegenerative disorders. In addition, more advanced DL methods and optimization methods can be explored to enhance the accuracy. Further improvements can be made by integrating multimodal data such as imaging and genetic information for better interpretation of results.

## Data Availability

The data that support the findings of this study are openly available in Kaggle repository at https://www.kaggle.com/datasets/rajmohnani12/huntington-disease-dataset, reference number^29^.

## Declarations

### Clinical trial registration

Not applicable. The research data used in this study were obtained from previously published sources, and no human subjects were involved or harmed.
